# Impact of Age and Insulin-Like Growth Factor-1 on DNA Damage Responses in UV-Irradiated Human Skin

**DOI:** 10.3390/molecules22030356

**Published:** 2017-02-26

**Authors:** Michael G. Kemp, Dan F Spandau, Jeffrey B. Travers

**Affiliations:** 1 Department of Pharmacology and Toxicology, Wright State University Boonshoft School of Medicine, Dayton, OH 45435, USA; mike.kemp@wright.edu; 2 Department of Dermatology, Indiana University School of Medicine, Indianapolis, IN 46202, USA; dspanda@iu.edu; 3 Department of Biochemistry and Molecular Biology Indiana University School of Medicine, Indianapolis, IN 46202, USA; 4 Dayton Veterans Affairs Medical Center, Dayton, OH 45428, USA

**Keywords:** Skin cancer, keratinocyte, insulin-like growth factor-1, UV light, DNA damage, DNA repair, DNA damage response, genomic instability, DNA replication, dermal wounding

## Abstract

The growing incidence of non-melanoma skin cancer (NMSC) necessitates a thorough understanding of its primary risk factors, which include exposure to ultraviolet (UV) wavelengths of sunlight and age. Whereas UV radiation (UVR) has long been known to generate photoproducts in genomic DNA that promote genetic mutations that drive skin carcinogenesis, the mechanism by which age contributes to disease pathogenesis is less understood and has not been sufficiently studied. In this review, we highlight studies that have considered age as a variable in examining DNA damage responses in UV-irradiated skin and then discuss emerging evidence that the reduced production of insulin-like growth factor-1 (IGF-1) by senescent fibroblasts in the dermis of geriatric skin creates an environment that negatively impacts how epidermal keratinocytes respond to UVR-induced DNA damage. In particular, recent data suggest that two principle components of the cellular response to DNA damage, including nucleotide excision repair and DNA damage checkpoint signaling, are both partially defective in keratinocytes with inactive IGF-1 receptors. Overcoming these tumor-promoting conditions in aged skin may therefore provide a way to lower aging-associated skin cancer risk, and thus we will consider how dermal wounding and related clinical interventions may work to rejuvenate the skin, re-activate IGF-1 signaling, and prevent the initiation of NMSC.

## 1. Introduction

Non-melanoma skin cancers (NMSCs) comprise the most common types of cancers in humans worldwide and originate from keratinocytes within the epidermal layer of the skin. In the United States alone, more than 2 million people are diagnosed with a NMSC each year [1,2]. The morbidity and high cost of treating NMSCs are a strain on both patients and the nation’s healthcare systems. These issues are particularly relevant for geriatric patients who make up the vast majority of NMSC cases [3] and who consume a high share of medical resources. Though there are a variety of approaches that can be employed to reduce NMSC incidence, novel interventions that are specifically targeted to older populations of people may therefore provide new and more effective ways of preventing skin carcinogenesis.

The single greatest risk factor for NMSC development is exposure to ultraviolet (UV) wavelengths of sunlight, which induce the formation of UV photoproducts in DNA. When not properly dealt with, these photoproducts may lead to mutations in genomic DNA that provide a growth advantage to epidermal keratinocytes and initiate a NMSC. The observed correlation between skin cancer and aging has traditionally been attributed to a lifetime of exposure to UVR that begins during childhood, which results in an accumulation of mutations that eventually drive tumorigenesis later in life. However, even in adults, sun avoidance and the application of sunscreens have been shown to reduce the incidence of actinic keratoses [4,5,6]. Thus, the initiation of UVR-induced carcinogenesis is not limited to youth and can occur throughout one’s lifetime. Nonetheless, the factors that affect the initiation of UV carcinogenesis may vary as a function of age. Indeed, the hypothesis that the altered physiology of geriatric skin may predispose keratinocytes in the epidermis to UVR-induced carcinogenesis has been considered and examined experimentally in recent years. In particular, the discoveries that the expression of insulin-like growth factor-1 (IGF-1) is lower in the skin of geriatric individuals than in young adults and that the IGF-1/IGF-1 receptor (IGF-1R) system regulates cellular responses to UVB has provided a paradigm shift in our understanding of aging-associated skin carcinogenesis [7,8].

In this review, we will therefore summarize how DNA photoproducts induced by UV wavelengths of light generate mutations in DNA and highlight the primary mechanisms by which cells respond to this DNA damage. This discussion will include an overview of nucleotide excision repair and DNA damage checkpoint signaling, which together allow cells to cope with a genome damaged by UV. Where appropriate, we will focus on published work that has addressed these issues in the context of aging and specifically within epidermal keratinocytes, which have the potential to become transformed and give rise to skin cancers. We will then review a growing body of literature that supports a role for the insulin-like growth factor (IGF-1) in keratinocyte responses to DNA damage and evidence that this system is de-regulated in geriatric skin. Lastly, we will discuss clinical interventions that can be employed to counteract this IGF-1-deficiency and the tumor-promoting environment of geriatric skin [9], which may provide a way to reduce skin carcinogenic risk in older patients.

## 2. UV-Induced DNA Damage Formation, Repair, and Checkpoint Signaling

### 2.1. UV-Induced DNA Photoproduct Formation

Photons of light with wavelengths in the range of 100–400 nm fall within the UV spectrum, which can be further subdivided as UVA (320–400 nm), UVB (280–320 nm), or UVC (100–280 nm) radiation. Though the sun emits UVR within all of these wavelengths, most UVC is absorbed by the Earth’s ozone layer. Thus, the UV wavelengths of sunlight that humans are typically exposed to on a daily basis are primarily composed of UVA (90%–95%) and UVB (5%–10%). A variety of cellular biomolecules absorb these wavelengths of light, including genomic DNA.

**Figure 1:**
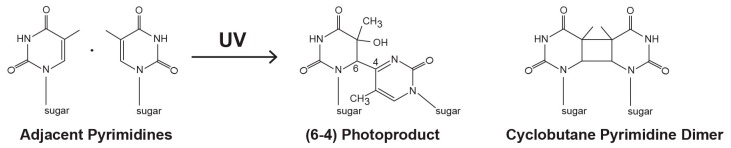
UV-induced photoproduct formation in DNA. The absorption of UV photons of light by adjacent pyrimidine nucleotides in DNA generates two major photoproducts, the pyrimidine (6-4) pyrimidone photoproduct [(6-4)PP] and the cyclobutane pyrimidine dimer (CPD). Though photoproduct formation between adjacent thymines is shown, (6-4)PPs and CPDs can also form to varying extents between adjacent cytosines and between cytosines and thymines.

The direct absorption of UV photons by DNA induces the formation of adducts between adjacent pyrimidine nucleotides [10,11], with cyclobutane pyrimidine dimers (CPDs) and pyrimidine (6-4) pyrimidone photoproducts [(6-4)PPs] being the most abundant (Figure 1). A variety of factors, including DNA sequence context and the specific energy of the UVR, affect the relative induction of CPDs and (6-4)PPs following UV exposure. Much of our understanding of cellular responses to UVR have been derived from studies using UVC light sources, and thus some caution is warranted in extrapolating these findings to physiological exposures of human skin to sunlight. Nonetheless, though DNA absorbs UVB light less efficiently than UVC, both CPDs and (6-4)PPs are generated by UVB wavelengths of light. UVA is also capable of inducing CPD formation by direct photon absorption [12,13] and via a recently characterized process termed chemiexcitation, in which reactive oxygen and nitrogen species induced by UVA combine to excite electrons in fragments of melanin that ultimately induce CPD production in genomic DNA [14,15]. Thus, both CPDs and (6-4)PPs are caused by UVA and UVB wavelengths of sunlight and are thought be biologically relevant to human disease risk.

These UV photoproducts are problematic to irradiated cells because the DNA lesions are potentially mutagenic and/or lethal. For example, cytosines and 5-methylcytosines within CPDs spontaneously deaminate to uracils and thymines, respectively, at a million-fold increased rate relative to the undamaged nucleotides [16,17,18,19]. This deamination is thus thought to be a major cause of the UV signature C→T transitions that are often found in skin cancer-associated p53 gene mutations [20]. Moreover, (6-4)PPs and CPDs are physical barriers to the progression of DNA and RNA polymerases during the processes of DNA replication and transcription [21,22,23], respectively. Though specialized DNA polymerases exist that can introduce nucleotides opposite damaged template DNA, these so-called translesion (TLS) polymerases frequently do so in an error-prone manner [24,25,26]. Replication fork stalling due to polymerase blockage may also lead to strand breakage that can give rise to chromosomal abnormalities, including translocations [27,28,29]. Lastly, UV-induced DNA damage can also cause cell death when essential gene products are unable to be transcribed by RNA polymerases and when stalled replication forks collapse to form catastrophic DNA double-strand breaks [23,30].

### 2.2. Removal of UV Photoproducts by Nucleotide Excision Repair (NER)

**Figure 2:**
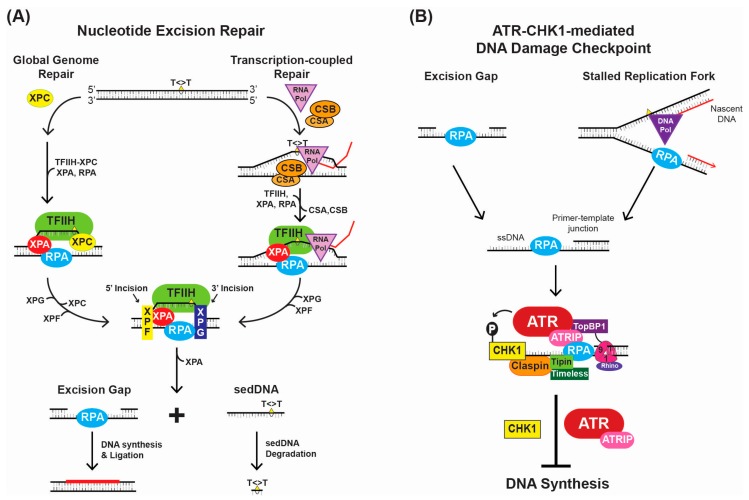
Schematic of nucleotide excision repair and the ATR-CHK1-mediated DNA damage checkpoint. (**A**) In nucleotide excision repair, UV photoproducts (denoted by T<>T and yellow triangles) are initially sensed through either the XPC-dependent global genome repair pathway or the CSA/CSB-dependent transcription-coupled repair pathway. Regardless of the mode of damage recognition, the TFIIH, RPA, and XPA function to verify the presence of the lesion and promote the assembly of the active repair machinery. The subsequent recruitment and incisions by the XPF and XPG endonucleases generate a ~30-nt-long excision gap and a small (~30-nt-long), excised, damage-containing DNA oligonucleotide (sedDNA). Filling in of the gap by a DNA polymerase and ligase and degradation of the sedDNA completes the repair reaction. (**B**) Unfilled excision gaps and DNA polymerase stalling at UV lesions generate regions of ssDNA that become bound by RPA and dsDNA/ssDNA primer-template junctions. These structures lead to the assembly of an active ATR-CHK1 signaling complex comprised of the RHINO-9-1-1 clamp and the ATR-activator TopBP1. In addition, RPA promotes the recruitment of the adaptor proteins Timeless-Tipin and Claspin, which function to specifically allow ATR to phosphorylate CHK1. The activation of these kinases leads to phosphorylation of a multiple downstream targets, many of which transiently prevent DNA synthesis in cells with UV-induced DNA damage.

Humans and other placental mammals possess a single system for removing UV photoproducts from genomic DNA known as nucleotide excision repair [31,32,33]. This repair system works by essentially cutting out the damaged bases from DNA in the form of a small DNA oligonucleotide approximately 30 nt in length. A schematic of NER is provided in Figure 2A. Depending on the mode of damage recognition, there are two ways in which NER can be initiated. In the transcription-coupled sub-pathway of NER (termed TC-NER), the stalling of an RNA polymerase at a UV photoproduct leads to the association of the Cockayne syndrome A and B proteins at the damage site, which then facilitate the recruitment of the core excision repair proteins that are necessary for damage excision. These factors include TFIIH (transcription factor II-H), RPA (replication protein A), and the XPA, XPF, and XPG proteins (xeroderma pigmentosum group A, F, and G). In contrast, in the global genomic repair pathway of NER (GG-NER) that operates throughout the genome, the damage recognition process requires XPC (xeroderma pigmentosum group C) instead of RNA polymerase. Nonetheless, regardless of the mechanism of damage recognition, the subsequent steps of NER are thought be identical and require the same five factors for damage excision. The multi-subunit protein factor TFIIH unwinds the DNA around the lesion to generate a repair bubble, and the XPA and RPA proteins facilitate the formation of a pre-incision complex that coordinates the actions of the structure-specific endonucleases XPF and XPG (XP group F and G), which cut the damaged strand of DNA at sites bracketing the lesion. This dual incision event therefore generates two reaction products, which include a small, single-stranded DNA (ssDNA) gap in the duplex DNA and a small, excised, damage-containing DNA oligonucleotide (sedDNA). The sedDNA is likely subsequently degraded by cellular nucelases, and the gap is filled in the by the actions of a DNA polymerase and ligase to complete the repair reaction [34].

Mutations in NER gene products give rise to both the disease xeroderma pigmentosum, which is characterized by a several thousand-fold increased risk of skin carcinogenesis, and to a neurodegenerative and premature aging disorder known as Cockayne syndrome [35,36]. Variations in NER gene product expression due to polymorphisms or other physiological factors within human populations are therefore expected to contribute to inter-individual differences in repair rates, skin aging, and in the propensity to develop skin cancers. Nonetheless, the removal of CPDs and (6-4)PPs by NER plays a critical role in preventing UV mutagenesis and maintaining cell and tissue viability following exposure to UVR.

### 2.3. Suppression of DNA Synthesis and Cell Cycle Progression by the DNA Damage Checkpoint

In addition to NER, cells have additional systems for detecting the presence of UV photoproducts throughout the genome that are thought to reduce the likelihood of introducing incorrect nucleotide opposite CPDs and (6-4)PPs during DNA replication and to provide additional time for damage removal by NER. These systems are termed DNA damage checkpoints and are comprised of protein kinases that regulate the activities of effector proteins that control DNA replication and cell cycle progression [37,38]. The two kinases most relevant to DNA replication-associated responses to UV-induced DNA damage are ATR (ataxia telangiectasia-mutated and rad3-related) and CHK1 (checkpoint kinase 1), which act as a part of a coupled signaling network [39,40,41] to transiently suppress DNA synthesis in UV-damaged cells by delaying the entry of damaged G1 cells into S phase, preventing new initiation events at replication origins [29,42,43,44,45,46,47], and slowing replication fork progression in UV-irradiated cells that are already within S phase [29,48,49].

The two most well-recognized signals for activation of the ATR-CHK1 signaling network are unfilled gaps generated by NER that subsequently get enlarged by exonucleolytic action [50,51,52] and the uncoupling of DNA polymerase and helicase activities at replication forks [53] (Figure 2B). Interestingly, both of these processes generate a common DNA structure defined by a long stretch of ssDNA and a dsDNA/ssDNA primer-template junction [54]. The ssDNA is thought to become coated by RPA [55,56], which is major ssDNA-binding protein in human cells [57,58]. The binding of RPA to ssDNA likely prevents nucleases from inappropriately cutting the ssDNA and generating potentially more detrimental double-stranded breaks in DNA. However, RPA also makes direct protein-protein contacts with numerous factors that facilitate the phosphorylation and activation of CHK1 by ATR. These interactions include associations with factors that recruit and activate both ATR and its canonical substrate CHK1. Thus, a number of studies have shown that RPA binding to the ATRIP (ATR-interacting protein) subunit of the ATR holoenzyme [59], the Rad9 component of the RHINO-9-1-1 clamp that is loaded onto the primer-template junction [60], and the ATR-activating TopBP1 and ETAA1 proteins [61,62,63,64] all play roles in promoting the activation of ATR. Similarly, through an interaction of RPA with the checkpoint mediator protein Tipin [65], CHK1 is recruited to ATR at sites of damage so that it can become phosphorylated and activated.

Once activated, ATR and CHK1 phosphorylate numerous protein targets that control DNA synthesis on UV-damaged templates [66,67,68,69]. Though the physiological significance of many of these substrates remains to be explored, some of the characterized checkpoint targets include the Cdc25 regulator of cyclin-dependent kinases (CDKs) necessary for S phase entry [70], the Treslin component of the replication initiation machinery [71,72,73], and additional factors important for replication fork elongation [74,75,76,77,78,79]. The relevance of this signaling pathway to the suppression of carcinogenesis is highlighted by studies showing that partial abrogation of ATR or CHK1 expression in mice increases the risk of tumorigenesis [39,80,81,82], including in the skin [83].

## 3. Effect of Aging on DNA Damage Responses in UV-Irradiated Human Epidermis

### 3.1. Effect of Age on UV Photoproduct Formation in the Epidermis

The process of aging can cause a number of changes to the morphology and physiology of skin [84,85,86,87], including a decrease in epidermal thickness and epidermal cell turnover. A decrease in the number of enzymatically active melanocytes in older individuals [88] may further contribute to a reduced ability of the epidermis to be protected from the induction of DNA damage by UV wavelengths of light. Thus, before examining how aging affects cellular responses to UV-induced DNA damage, it is important to understand how aging impacts UV photoproduct formation in the epidermis of the skin. However, examination of this issue has been rather limited.

Nonetheless, one relevant study used the ^32^P-postlabeling method to quantify the induction and subsequent removal of a number of UV photoproducts from the epidermis of 30 human subjects of diverse age and skin type [89]. The methodology involved exposing previously unexposed participant buttock skin to 400 J/m^2^ of solar simulating radiation, excising a small punch biopsy of the area, and then purifying the genomic DNA from the epidermis. This genomic DNA was then treated with a panel of nucleases to produce nucleoside-3′-phosphates that were subsequently 5′-labeled with T4 polynucleotide kinase and detected as trinucleotides (containing an unmodified 5′-thymidine) by high performance liquid chromatography. The use of photoproduct standards provided the investigators the means to quantify the abundance of TT<>T and TT<>C trinucleotides containing CPDs and (6-4)PPs within the subjects’ epidermal genomic DNA.

Interestingly, when the authors classified the subjects by age, the levels of all four of these adduct-containing damages were found to be higher in individuals over the age of 50 than in individuals under the age of 50 [89], though only the CPD-containing TT<>Cs reached a statistically significant difference. Nonetheless, using a multivariate regression analysis, the authors concluded that age was a more important factor than skin type in determining UV photoproduct levels and that aging one year caused an increase of roughly 1 CPD and 0.1 (6-4)PP per 10^7^ nucleotides. As will be discussed in greater detail below, several other studies that have addressed UV photoproduct levels in the epidermis as a function of subject age have typically only done so in the context of DNA repair and have not provided sufficient information regarding the levels of initial DNA damage caused by UV exposure. Consideration of this issue in future work may therefore provide a more complete picture regarding how skin aging affects both the generation of UV photoproducts and the subsequent cellular responses. The use of deep sequencing technologies such as Damage-seq [90] and related approaches to map UV photoproduct formation at specific genomic locations [91,92,93,94,95] would provide valuable information regarding how photoproduct induction across the genome changes as people age. It may also be advantageous to examine how proliferating keratinocytes in the basal layer of the epidermis, which are the cells that are capable of undergoing mutagenesis and transformation to give rise to skin tumors, are specifically affected by UV irradiation in aged skin.

### 3.2. Effect of Age on UV Photoproduct Repair in the Epidermis

Though there has long been interest in understanding the association between aging and the repair of UV photoproducts [96], many previous studies have yielded conflicting results and have been limited to fibroblasts or lymphocytes that were cultured and studied in vitro [97]. Measurements of UV photoproduct repair within the epidermis of the skin in situ may therefore be considered to be more physiologically relevant, and fortunately a number of studies have taken this approach. One such early investigation [98] exposed the skin of volunteers between the ages of 23 and 69 to 1 MED (minimal erythemal dose) of UV light with a sunlamp that emits wavelengths between 280 and 400 nm and then isolated the epidermis from punch biopsies at various time points following UV exposure. A classical DNA repair assay utilizing the pyrimidine dimer-specific Micrococcus luteus UV-endonuclease [99] was then employed to detect the presence and subsequent time-dependent loss of CPDs from epidermal genomic DNA. This report showed that whereas it took approximately 10.3 h for subjects within their 20 s to remove 50% of CPDs from genomic DNA, the same degree of CPD repair took 19.3 h among individuals over the age of 65 [98]. Thus, this study supported the concept that the removal of UV photoproducts is impaired in the skin of geriatric individuals relative to younger subjects.

In addition to quantifying DNA adduct levels immediately following UV exposure, the study employing the ^32^P-postlabeling and HPLC method described earlier [89] also examined the loss of the damaged nucleotides from epidermal genomic DNA at 24 and 48 h after irradiation. This study found that patients over the age of 50 had more CPD-containing TT<>T trinucleotides remaining in their epidermal genomic DNA 24 h after UV exposure than subjects under the age of 50, though this difference was no longer present by 48 h. However, this difference in photoproduct loss from genomic DNA was not observed for CPD-containing TT<>Cs. Thus, there may be some degree of sequence specificity regarding the repair of specific UV photoproducts that impact overall repair efficiency in the epidermis. In addition, because this study stratified patients into two rather broad groups that were either younger or older than 50 years of age, this limited data set may fail to adequately detect age-dependent changes in DNA repair.

Nonetheless, the notion that nucleotide excision repair of UV photoproducts occurs at a slower rate in the skin of older individuals was supported by a more recent study that compared CPD removal rates between subjects in their 20 s and 70 s, all of whom had a type III or IV skin type [100]. These authors used an anti-CPD antibody and both immunocytochemistry and immunoslot blot analysis of genomic DNA from the epidermis of the upper arm to quantify the loss of CPDs over the course of up to two weeks. Whereas CPDs were completely gone from the epidermis of the younger subjects within 4 days following exposure to 0.5 MED (with a light source that emits wavelengths between 275 and 410 nm), approximately 50% of the CPDs remained in the epidermis of the skin at this time point in the geriatric individuals. In these older individuals, complete CPD removal took up to 2 weeks to take place. Whether or not this slower loss of CPDs was due solely to an NER defect, or also to reduced epidermal cell turnover was not determined.

The three studies described above examined the loss of UV photoproducts from whole epidermis. Though keratinocytes throughout the different layers of the epidermis will contain DNA damage following UV exposure, the effect of UV on the proliferating cells within the basal layer is potentially most relevant to skin carcinogenesis. Using anti-CPD and anti-Ki67 antibodies to stain replicating keratinocytes containing UV photoproducts, a more recent study showed that whereas skin from subjects between the ages of 20 and 28 years displayed very few replicating keratinocytes containing DNA damage 24 h after exposure to 350 J/m^2^ of UVB (indicative of complete CPD removal by nucleotide excision repair), geriatric skin from subjects >65 years of age displayed many such cells [8]. As will be described below, this abnormal response to UVB exposure in geriatric skin was found to be correlated with an increase in senescent fibroblasts in the dermis of the skin and with an abrogated production of insulin-like growth factor (IGF-1).

Together, these several studies indicate that the efficiency by which epidermal keratinocytes in human skin are able to remove CPDs may decrease as people age. However, it should be noted that the different approaches for measuring CPD repair that were used in the studies above have a number of limitations that are relevant to UV-induced mutagenesis and skin carcinogenesis. The first issue is that these studies have generally focused on CPDs and ignored (6-4)PPs, which are also capable of introducing mutations into genomic DNA. Although (6-4)PPs are generally repaired at a much faster rate than CPDs, this repair largely takes place within the same time frame as the bulk of ATR-CHK1 signaling (during the first 4 h post-UV). Thus, defects in (6-4)PP removal, ATR-CHK1 signaling, and in the suppression of chromosomal DNA synthesis after UV could in principle be associated with mutagenesis that gives rise to skin cancers. Furthermore, the accuracy of assays for measuring CPD removal, which in many studies takes place on the time scale of days, may be complicated by effects of cell proliferation, epidermal cell turnover, and apoptosis. These processes may dilute CPD content within genomic DNA in a DNA repair-independent manner, and this complication therefore affects the accuracy of CPD quantitation as a reliable measure of nucleotide excision repair capacity in the skin. Thus, the application of novel technologies, including assays that directly detect the sedDNA products of nucleotide excision repair [101,102,103,104,105], may be advantageous in the future for quantifying DNA repair capacity as a function of age following UV exposures and for correlating these factors to skin carcinogenic risk.

### 3.3. Effects of UVR on DNA Synthesis and DNA Damage Checkpoints in the Epidermis

Whereas a number of studies have explored UV photoproduct formation and repair in human skin in vivo, much less is known regarding the effects of UVR on epidermal keratinocyte DNA synthesis and DNA damage checkpoint signaling in human epidermis. Nonetheless, an early study employed tritiated thymidine injection into the skin of human subjects following UVR exposure to monitor how UVR affects both normal, chromosomal DNA replication and DNA synthesis associated with DNA repair [106]. These two types of replication have been classically defined by autoradiographic microscopy as cells with either heavy labeling throughout the cell or with sparse labeling at purported sites of DNA repair, respectively. Interestingly, the study observed that within 3–5 h after UV exposure, the percentage of basal keratinocytes undergoing chromosomal DNA synthesis decreased from approximately 5% to 2.5%–2.8% [106]. By 24 h after UV exposure, the percentage of basal cells performing DNA replication recovered to the level of unirradiated skin. Thus, this apparent transient inhibition of DNA synthesis may represent an active in vivo DNA damage checkpoint similar to that reported in cultured cells exposed to UVR in vitro. Whether ATR and CHK1 are responsible for this inhibition of DNA synthesis in UV-irradiated human skin is not known. Though a recent study demonstrated that the canonical ATR-dependent phosphorylation of CHK1 can be observed within the epidermis of human skin explants exposed to UVR ex vivo [107], previous studies of ATR and CHK1 in the context of skin have been largely restricted to mouse models [83,108]. However, one study using human foreskin explants observed an increase in cyclin B-positive basal keratinocytes 24 h after a sub-erythemal dose of UVR, which is indicative of a G2 checkpoint [109]. Moreover, this study observed that the topical application of caffeine, which is a known inhibitor of ATR kinase activity [110], abrogated this UV-induced G2 checkpoint [109]. This finding suggests that ATR may indeed play a role in cell cycle checkpoints in UV-irradiated skin. Thus, future analyses of ATR-CHK1 signaling and DNA synthesis in UV-irradiated human skin in vivo, and the examination of these responses as a function of patient age, may therefore provide new clues into the early events of UV skin carcinogenesis in humans.

## 4. Effect of Aging on Insulin-Like Growth Factor-1 (IGF-1) Production in the Skin

### 4.1. Epidermal Keratinocyte IGF-1 Receptor (IGF-1R) Activation Is Altered in Aged Skin

As the most abundant cell type in the epidermis, keratinocytes are the major target of UVR and are also the cell type of origin for the development of NMSCs. Though keratinocytes are capable of responding to UV in a cell autonomous manner, interactions of keratinocytes with other cell types within the skin are also expected to influence keratinocyte responses to UVR. An important goal of skin carcinogenesis research is therefore to understand how the physiological environment of the skin contributes to NMSC development. Given that many aspects of skin biology change as human age [84,85,86], an additional issue to consider is whether these changes affect the cellular response of keratinocytes to UV-induced DNA damage.

**Figure 3:**
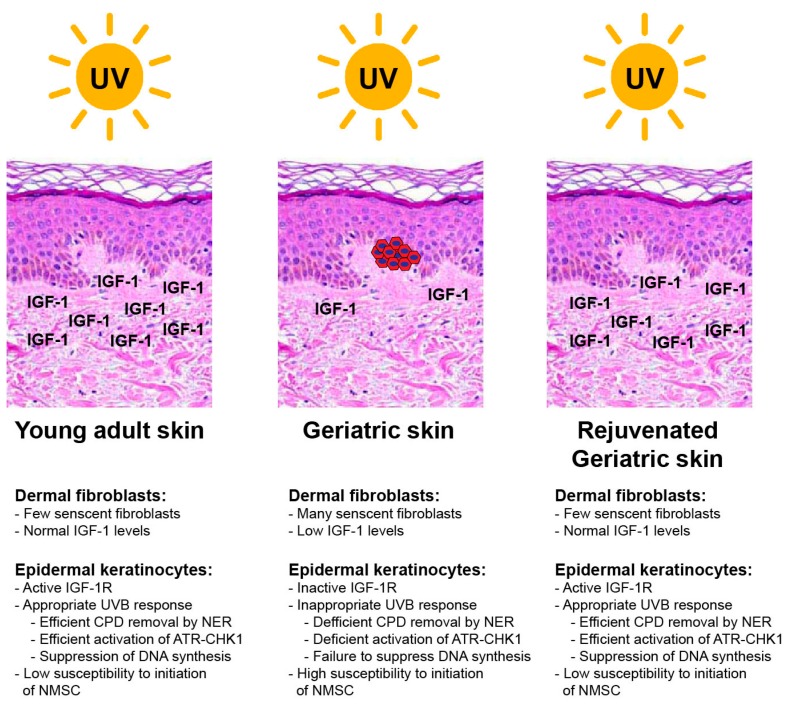
Model for the effects of age and IGF-1 status on fibroblast and keratinocyte behavior in UVB-irradiated human skin. (Left panel) In young adult skin, dermal fibroblasts produce IGF-1, which leads to active IGF-1Rs on keratinocytes in the epidermis. Thus, when these keratinocytes are exposed to UVB, they carry out an appropriate response, which includes efficient nucleotide excision repair (NER), activation of ATR-CHK1 signaling, and the suppression of DNA synthesis. Together, this appropriate response is associated with a low susceptibility to NMSC initiation. (Middle panel) In contrast, in geriatric skin containing many senescent fibroblasts, the reduced production of IGF-1 leads to inactive IGF-1Rs in epidermal keratinocytes. Exposure of these cells to UVB leads to an inappropriate response that includes deficiencies in NER, ATR-CHK1 signaling, and in the suppression of DNA synthesis, which may culminate in mutagenesis and a higher susceptibility to initiate NMSC. (Right panel) Geriatric skin treated with dermal rejuvenation intervention behaves similar to that of young adult skin.

Through the regulation of various intracellular signaling pathways that control cell proliferation and other cellular phenotypes, growth factors play fundamental roles in general cell biology, including within the epidermis of the skin. Local paracrine signaling in particular may affect keratinocyte growth and response to exogenous stress. One such factor of relevance to the epidermis and its response to UV-induced DNA damage is insulin-like growth factor-1 (IGF-1). In human skin, keratinocytes express the IGF-1 receptor (IGF-1R) but do not produce IGF-1 [111,112,113]. Instead, the major provider of IGF-1 to epidermal keratinocytes are fibroblasts in the underlying dermis [111,112,113]. The stimulation of the IGF-1R by IGF-1 activates a variety of intracellular signaling pathways, including the PI3K/AKT and MAPK networks [114]. As dermal fibroblasts age in vitro and become senescent, their ability to produce IGF-1 becomes reduced [8,115,116]. This in vitro finding has physiological relevance in vivo, as both increases in fibroblast senescence and decreases in IGF-1 production have been observed in the skin of geriatric patients over the age of 65 relative to the skin of younger subjects in their 20 s [116]. Consistent with the idea that dermal production of IGF-1 impacts the activation status of the IGF-1R in keratinocytes, an examination of IGF-1R phosphorylation as a measure of its activation revealed it to be decreased in epidermal keratinocytes of geriatric skin in comparison to that in young adult skin [8]. These differences between young adult and geriatric skin regarding dermal fibroblast and epidermal keratinocyte function are summarized in Figure 3, and will be described in more detail below.

### 4.2. The IGF-1/IGF-1R System Affects Cell Fate Following Exposure to UVR

Extensive DNA damage caused by UVR may lead cells to undergo either apoptosis or senescence [117,118]. Both of these processes limit the ability of damaged cells that potentially contain UVR-induced gene mutations from undergoing continued proliferation, which in the context of the epidermis may otherwise lead to tumorigenesis [119,120]. Studies with primary neonatal foreskin keratinocytes cultured in vitro demonstrated that the specific inactivation of the IGF-1R via withdrawal of IGF-1 ligand from the culture medium predisposed UVB-irradiated cells to undergo apoptosis [8,121]. To mimic the physiological environment of the skin, additional in vitro experiments were carried out using conditioned medium from fibroblasts depleted of IGF-1 via RNA interference, from fibroblasts induced to undergo senescence via oxidative stress or serial passaging (which resulted in reduced IGF-1 expression), and from fibroblast-derived conditioned medium supplemented with anti-IGF-1 antibody [8,122]. The use of each of these conditioned mediums failed to protect keratinocytes from undergoing apoptosis following exposure to high dose UVB. These results therefore validated the hypothesis that fibroblast-derived IGF-1 regulates keratinocyte responses to UVB and suggested that a reduction in IGF-1 expression by fibroblasts in geriatric skin may alter the fate of epidermal keratinocytes to UVB-induced DNA damage.

When UV-induced DNA damage is less extensive, cells may undergo a permanent growth arrest known as senescence [117,118]. These cells remain viable and can contribute to tissue integrity in vivo but are incapable of further cell division. Interestingly, when primary keratinocytes were exposed to senescence-inducing doses of UVB in vitro, cells that had been deprived of IGF-1 were found to be less likely to undergo senescence [8,123]. Co-staining of these cells for CPDs and the proliferation marker Ki67 revealed that these cells continued to proliferate in vitro in the presence of DNA damage. Furthermore, this in vitro finding with cultured keratinocytes also held true in the context of the epidermis from geriatric skin that displayed reduced IGF-1 expression in vivo [8]. Thus, the epidermis from the UVB-irradiated skin of subjects greater than 65 years old were found to contain significantly more proliferating keratinocytes with unrepaired CPDs 24 h after UVB exposure than skin from individuals 20–28 years old [8], which instead possessed few such cells. To show that this response was dependent on IGF-1, recombinant IGF-1 was injected into the skin of geriatric patients prior to UVB exposure and was found to lead to a significant reduction in CPD+/Ki67+ double positive cells 24 h following irradiation of the skin. Thus, the presence of IGF-1 in the skin and active IGF-1Rs in keratinocytes therefore appear to be required to prevent keratinocytes with unrepaired CPDs within the basal layer of the epidermis from continuing to proliferate.

### 4.3. The Removal of UV-Induced CPDs Is Affected by IGF-1R Status in Human Keratinocytes

To better understand the link between IGF-1 and UV photoproduct removal in human keratinocytes, additional studies were recently carried out using primary and telomerase-immortalized human adult or neonatal foreskin-derived keratinocytes that were cultured in vitro [122,124]. In these experiments, the rate of CPD removal from genomic DNA was monitored by either immunofluorescence microscopy or immunoslot blot analysis with anti-CPD antibody under conditions in which the IGF-1R was inactivated by either direct IGF-1 withdrawal [122,124], the use of a small molecule inhibitor of the IGF-1R [124], or fibroblast-conditioned medium supplemented with anti-IGF-1 antibody [122]. Regardless of the mode of IGF-1R inhibition, the rate of CPD removal was found to be significantly slowed. These in vitro findings were also expanded upon and confirmed through experiments with human skin ex vivo and in vivo [124]. Experiments with human abdominoplasty skin treated topically with either DMSO vehicle or an IGF-1R inhibitor prior to UVB irradiation and the subsequent isolation of basal keratinocytes revealed that the removal of CPDs was partially abrogated when the IGF-1R was inhibited [124]. Moreover, human skin grafted onto the backs of SCID/NOD mice and treated topically with an IGF-1R inhibitor was shown to be associated with a high number of CPD+/Ki67+ basal keratinocytes 24 h after UVB exposure [124], which mimics the phenotype of geriatric skin in human subjects.

Studies with cultured keratinocytes in vitro demonstrated that disruption of IGF-1R signaling was associated with a reduction in the expression of the NER factors XPC and XPF/ERCC4 at both the level of mRNA and protein [124]. These findings indicate that de-regulation of the IGF-1/IGF-1R system during aging may lead to reduced NER gene expression that subsequently prevents keratinocytes from efficiently removing UV photoproducts from genomic DNA. This altered rate of repair may therefore increase the risk of mutagenesis and skin carcinogenesis. Given that inter-individual variability in NER has long been thought to impact skin carcinogenic risk, it will therefore be interesting to determine whether the expression of XPC and XPF/ERCC4 is reduced in the epidermis of geriatric skin relative to the skin of younger individuals.

### 4.4. Disruption of ATR-CHK1 Kinase Signaling and the Suppression of DNA Synthesis in Keratinocytes with Inactive IGF-1Rs

Though nucleotide excision repair is a major system that protects keratinocytes from DNA damage and associated mutagenesis associated with UV exposures, it is not the only protective barrier to cancer initiation. Cells also possess various DNA damage signaling pathways that sense DNA damage and transiently arrest cell cycle progression and DNA synthesis to provide additional time for DNA repair. Together, DNA repair and DNA damage checkpoint signaling therefore limit mutagenesis and carcinogenesis. As described above, the ATR and CHK1 kinases play a major role in regulating these various DNA damage responses following UVB exposure, and partial disruption of their activities is linked to tumorigenesis [39,80,81,82,83].

Two recent studies have examined how the activation status of the IGF-1R affects ATR-CHK1 signaling following UVB exposure in cultured keratinocytes [107,122]. Both studies reported a reduction in the phosphorylation of the canonical ATR substrate CHK1 after UVB exposure when the IGF-1R was inactivated by either IGF-1 withdrawal or pharmacological inhibition. The relevance of these findings to keratinocytes in intact human skin was further demonstrated in human skin explants treated with an IGF-1R inhibitor prior to UVB treatment ex vivo, and similarly demonstrated a reduction in UVB-induced CHK1 phosphorylation. Because the ATR-CHK1 signaling pathway targets several components of the DNA synthesis machinery, BrdU immunodot blot analysis was used to monitor the kinetics of DNA replication following UVB exposure in cultured keratinocytes in vitro. To minimize mutagenesis, cells damaged by low, non-toxic doses of UV or related chemical carcinogens transiently suppress DNA synthesis for several hours before resuming a normal rate of DNA replication [29,42,44,45,46,47,125]. This response is known to be abrogated in cells with deficient ATR-CHK1 signaling [42,125]. Interestingly, and consistent with biochemical analyses of the ATR-CHK1 signaling network, inhibition of the IGF-1R was shown to partially abrogate this suppression of DNA synthesis after UVB exposure in cultured keratinocytes [107]. This checkpoint disruption may lead to increased mutagenesis, and additional experiments will be necessary to test this hypothesis. Moreover, given that ATR signaling has been linked to several other DNA damage responses in UV-irradiated cells, including apoptosis [126,127,128,129,130], senescence [131,132], and nucleotide excision repair during S phase [133,134], it is possible that the previously reported defects in these processes in keratinocytes with an inactive IGF-1R may be due in part to altered ATR kinase signaling.

The observation that both nucleotide excision repair and ATR-CHK1 signaling are disrupted in keratinocytes with inactive IGF-1Rs indicates that both phenotypes may be caused by a single, common defect in DNA metabolism. Indeed, both systems utilize the ubiquitous DNA metabolic protein RPA, which plays critical roles in DNA replication, repair, and recombination [55,56,57,58]. In the context of the cellular response to UVR, RPA facilitates photoproduct recognition and coordinates the recruitment and/or enzymatic activities of a number of proteins during both nucleotide excision repair [58,135,136,137,138,139,140,141,142,143,144,145,146,147] and ATR-CHK1 signaling [59,60,61,65]. Though RPA normally becomes enriched in the chromatin fraction of keratinocytes within an hour after DNA damage induction by UVB where it can carry out NER and checkpoint signaling, a recent study found that this response was partially disrupted when the IGF-1R was inactivated [107]. Furthermore, the basal level of chromatin-associated RPA was observed to be elevated in IGF-1R inhibitor-treated cells prior to UVB exposure, which may indicate the presence of ssDNA due to endogenous replicative stress. Thus, imbalances in growth factor signaling may generate cellular stress that subsequently interferes with the ability of keratinocytes to properly respond to UV.

Though RPA is generally thought to be an abundant nuclear protein, the fact that it functions in so many diverse DNA metabolic processes may become problematic under conditions in which its availability becomes limiting. Consistent with this notion, several recent studies have suggested that “RPA exhaustion” is a frequent problem in cells undergoing extensive replicative stress [134,148,149,150,151], including following UV exposure. Thus, it is possible that an insufficient supply of RPA in keratinocytes stressed by IGF-1R inactivation may lower the pool of RPA available for NER and ATR-CHK1 signaling. However, this hypothesis awaits experimental validation.

## 5. Dermal Wounding as a Preventive Approach for NMSC

There has long been an interest in the use of cosmetic dermal rejuvenation approaches to create more youthful-appearing skin [152,153]. These rejuvenation methods are diverse and include approaches such as dermabrasion and fractionated laser resurfacing [154]. These skin rejuvenation therapies are thought to induce a wounding response in dermal fibroblasts that ultimately stimulates the production of new collagen synthesis by fibroblasts in the skin. Indeed, both dermabrasion and fractionated laser resurfacing have been shown to decrease the percentage of senescent fibroblasts in the skin and to result in an increase in collagen expression in geriatric skin [116,155].

Given that an increase in dermal fibroblast senescence in geriatric skin relative to young skin is correlated with a decrease in the expression of IGF-1 [8], the effect of these dermal wounding strategies on IGF-1 expression has therefore been examined over the past few years. Importantly, both rejuvenation methodologies were shown to result in an increase in IGF-1 expression in the skin of geriatric subjects [116,155] (Figure 3, right panel). Clinical studies were therefore carried out to compare the response of non-rejuvenated and rejuvenated skin of geriatric subjects to UVB by examining the presence of proliferating (Ki67+) keratinocytes containing unrepaired CPDs 24 h after UV exposure. Interestingly, and similar to that observed in the skin of young individuals, regions of skin that were rejuvenated by dermabrasion or fractionated laser resurfacing displayed a near complete absence of CPD+/Ki67+ keratinocytes [116,155]. Whether these methods allow keratinocytes to better utilize additional protective DNA damage responses, such NER and the ATR-CHK1 checkpoint signaling cascade, is currently unknown. It will therefore be interesting to determine whether these rejuvenation strategies elevate the expression of XPC and XPF/ERCC4 [124] and promote proper RPA function and ATR-CHK1 signaling in proliferating keratinocytes [107]. As described above, the replication of UV-damaged DNA has the potential to introduce mutations that give rise to cancer. Thus, identifying all of the ways in which geriatric skin behaves differently than young skin in response to UVB and characterizing the processes that can be modulated by skin rejuvenation approaches may be useful for better understanding the origin of NMSCs.

Nonetheless, these novel findings suggest that wounding therapies have the potential to be useful in preventing the initiation of NMSCs in geriatric patients [9,156]. It will therefore be interesting to determine whether there are differences between non-rejuvenated and rejuvenated skin in the emergence of keratinocytes with UV signature mutations following repeated exposures to UVB light. Long-term follow-up of patients with regions of rejuvenated skin will therefore shed important insights on this issue.

## 6. Conclusions

The fact that the majority of NMSCs occur in patients over the age of 60 indicates that the physiology of aged skin may contribute to the risk of UVB-induced skin carcinogenesis. As summarized here, the skin of geriatric individuals is characterized by an increase in dermal fibroblast senescence and a corresponding decrease in IGF-1 production. This phenotype is associated with the decreased activation of the IGF-1R in epidermal keratinocytes and in altered cellular responses to UVB-induced DNA damage, including defects in UV photoproduct removal rate by NER, ATR-CHK1 kinase signaling, and in the suppression of DNA synthesis following UVB exposure. Together these altered responses to DNA damage increase the likelihood of mutagenesis and NMSC development. Fortunately, these negative outcomes may be counteracted by dermal wounding methods that rejuvenate the skin and restore the IGF-1/IGF-1R system to that found in young skin. Thus, dermal wounding has the potential to become a cost effective method for preventing NMSC initiation in aging populations.
